# Non-Coding RNAs and SARS-Related Coronaviruses

**DOI:** 10.3390/v12121374

**Published:** 2020-12-01

**Authors:** Hanna Henzinger, Dominik A. Barth, Christiane Klec, Martin Pichler

**Affiliations:** 1 Comprehensive Cancer Center Graz, Research Unit of Non-Coding RNAs and Genome Editing, Department of Internal Medicine, Division of Clinical Oncology, Medical University of Graz, 8036 Graz, Austria; hanna.henzinger@stud.medunigraz.at (H.H.); dominik.barth@medunigraz.at (D.A.B.); christiane.klec@medunigraz.at (C.K.); 2 Department of Experimental Therapeutics, The University of Texas MD Anderson Cancer Center, Houston, TX 77030, USA

**Keywords:** SARS, COVID, coronavirus, non-coding RNA, miRNA, siRNA, long non-coding RNA, therapy

## Abstract

The emergence of SARS-CoV-2 in 2019 has caused a major health and economic crisis around the globe. Gaining knowledge about its attributes and interactions with human host cells is crucial. Non-coding RNAs (ncRNAs) are involved in the host cells’ innate antiviral immune response. In RNA interference, microRNAs (miRNAs) may bind to complementary sequences of the viral RNA strand, forming an miRNA-induced silencing complex, which destroys the viral RNA, thereby inhibiting viral protein expression. There are several targets for human miRNAs on SARS-CoV-2’s RNA, most of which are in the 5’ and 3’ untranslated regions. Mutations of the viral genome causing the creation or loss of miRNA binding sites may have crucial effects on SARS-CoV-2 pathogenicity. In addition to mediating immunity, the ncRNA landscape of host cells further influences their susceptibility to virus infection, as certain miRNAs are essential in the regulation of cellular receptors that are necessary for virus invasion. Conversely, virus infection also changes the host ncRNA expression patterns, possibly augmenting conditions for viral replication and dissemination. Hence, ncRNAs typically upregulated in SARS-CoV-2 infection could be useful biomarkers for disease progression and severity. Understanding these mechanisms could provide further insight into the pathogenesis and possible treatment options against COVID-19.

## 1. Introduction

Following the outbreak of the coronavirus disease 2019 (COVID-19) pandemic in 2019, there has been a lot of research concerning the attributes of severe acute respiratory syndrome coronavirus 2 (SARS-CoV-2), its pathogenic mechanisms, and potential treatments against COVID-19.

SARS-CoV-2 is a single-stranded, positive RNA virus that is closely related to SARS-CoV, the causative agent of SARS, which already led to a pandemic in 2003 [1,2].

The RNA of both viruses is around 30 kb long and consists of 14 open reading frames (ORFs) that encode for a total of four structural and 16 non-structural proteins (NSPs), as well as a capped leader sequence (5’ untranslated region (UTR)) and a polyadenylated terminus (3’ UTR) [3,4,5].

SARS-CoV-2 and SARS-CoV both use one of their structural proteins, the spike (S) protein, to mediate cell invasion. Human angiotensin-converting enzyme 2 (ACE2) serves as a receptor for the S protein [4,6], while type II transmembrane serine protease (TMPRSS2), which is responsible for the cleavage and activation of the S protein, comprises a necessary co-receptor for the completion of the infection process [7].

The S protein sequences of the two viruses are 76% identical [8], with SARS-CoV-2’s S protein gene having additional nucleotides that form a furin-like cleavage site, which is considered to be responsible for the higher infectivity of the virus compared to other similar coronaviruses [2,9].

Upon infection, the most common symptoms that can be observed in COVID-19 include fever and respiratory failure, although gastrointestinal and neurological symptoms have also been observed [10]. SARS-CoV-2 mainly targets the lungs, where, in severe cases, infection results in acute respiratory distress syndrome (ARDS). ARDS leads to diffuse alveoli damage (DAD) and often correlates with an excessive release of pro-inflammatory cytokines, called a “cytokine storm” [11].

The aim of this review was to give a comprehensive overview of the current knowledge about the involvement of non-coding RNAs (ncRNAs), in particular, micro RNAs (miRNAs), small interfering RNAs (siRNAs), and long non-coding RNAs (lncRNAs) in the pathogenesis of SARS-CoV-2 and the antiviral immune defense mechanisms of the host. To date, treatment options for SARS-CoV-2 infection remain scarce [12]. Considering SARS-CoV-2 as an RNA virus, the present work also aimed to shed a light on possible therapeutic applications of ncRNAs in antiviral therapies and to encourage further research in this field.

## 2. Methods

This paper was based solely on literature research using the “PubMed” database. The platform was searched using various combinations of the terms “non-coding RNA,” “ncRNA,” “SARS,” and “Covid” and a filter for free full texts was applied. Studies in which ncRNAs were only used as research tools or where SARS only served as an example in topics regarding other viruses were not included, as this review was particularly focused on the role of non-coding RNAs in the interactions between human cells and SARS-related coronaviruses.

## 3. RNA Interference

RNA interference (RNAi) mediated by siRNAs or miRNAs is an important immunity mechanism in plants and invertebrates. In mammals, however, the dominating antiviral innate immune response is the interferon (IFN)-mediated response and the existence of an additional RNAi response in mammals has only been verified recently [13,14]. While siRNA-mediated RNA interference is only present in embryonic stem cells of mammals [15], RNAi mediated by miRNAs directly bind to the viral genome or messenger RNAs (mRNAs) of viral genes, thereby limiting viral replication and mitigating pathogenicity, which can also be observed in differentiated adult host cells [16,17].

### 3.1. Antiviral Interactions between Host miRNAs and Viral RNA

**Figure 1:**
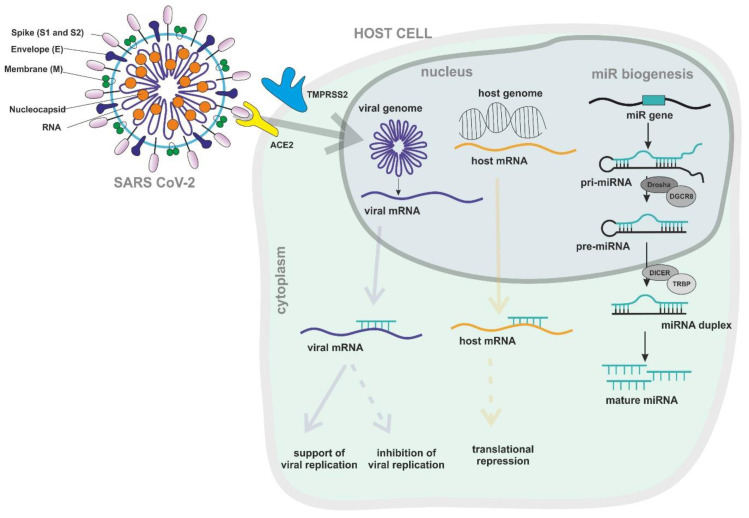
Schematic representation of severe acute respiratory syndrome coronavirus 2 (SARS-CoV-2) viral structure and the consequences of infection for the host cell concerning miRNA-mediated cellular regulation. mRNA: messenger RNA, pre-miRNA: precursor microRNA, pri-miRNA: primary microRNA.

In general, miRNA–virus interactions can result in two different situations: the repression of viral translation, which inhibits viral replication, or stabilization of the viral RNA, which, in contrast, enhances viral replication [16,18]. In RNAi, the former is the case, as miRNAs acting in the miRNA-induced silencing complex (miRISC) bind to viral mRNA and, if there is complete complementarity, induce mRNA decay. If the pairing between the viral mRNA and host miRNA is imperfect, the mRNA will not be degraded but translation will still be inhibited, resulting in the ability of one single miRNA to target multiple mRNAs [19,20]. miRNA binding sites in viral RNAs are typically located in the 5’ and 3’ UTRs [3,16,21] but have also been found in coding regions, e.g., in the genome of influenza A [22] and enterovirus 71 [23]. Since positive-strand RNA virus genomes are structurally identical to mRNAs, they might be regulated by miRNAs in a similar way [16] (Figure 1).

Hosseini et al. [24] recently identified seven targets of miRNA in the genome of SARS-CoV-2. Originally, there were ten targets, but three of them were lost because of conserved mutations. Among the human miRNAs that are able to bind to SARS-CoV-2 encoded transcripts, thereby mediating immunity, are miR-574-5p, miR-214, miR-17, miR-98, miR-223, and miR-148a [24].

Furthermore, Fulzele et al. [25] found a total of 873 human miRNAs targeting the SARS-CoV-2 strain, 558 of which are also directed against sequences in the SARS-CoV genome. According to their research, the miRNAs that had the highest target score and are therefore most likely to have actual target sites in SARS-CoV-2 were miR-15a-5p, miR-15b-5p, miR-30b-5p, miR-409-3p, miR-505-3p, and miR-548d-3p [25].

However, computational predictions of miRNA binding sites should be viewed with caution, as results often fail to be verified experimentally [26,27,28].

This high false-positive rate of miRNA target prediction has been described frequently, yet it can be narrowed down using certain approaches, such as multi-targeting, integration of existing experimental evidence, or the use of algorithms designed to refine the results of miRNA target searches [27,29]. It may also be beneficial to consider conditions specific to the research question (e.g., excluding potential targets of all miRNAs that are not expressed in cells prone to SARS-CoV-2 infection [24]).

Since the studies by Hosseini et al. [24] and Fulzele et al. [25] have taken such measures to narrow down their results, their findings still appear promising and should be taken into account in further research on COVID-19 pathogenesis and potential RNAi therapeutics.

### 3.2. Evasion of miRNA-Mediated RNAi in the Untranslated Regions

The UTRs at both ends of the viral RNA serve as control elements in its replication, transcription, and translation [30], and might influence the evasion of host RNA decay. The 5′ and 3′ UTRs include binding sites for host miRNAs and RNA-binding proteins (RBPs). While the binding of host-derived miRNA to a viral RNA usually results in degradation of the RNA, certain RBPs binding to transcripts of the 5′ UTR, such as CUG-binding protein (CUG-BP) and trans-active response DNA binding protein (TARDBP), increase the translation of viral proteins [3]. For example, in a SARS-CoV-2 variant, the binding site for CUG-BP turns into a TARDBP binding site via a change of “C” to “U” on position 241, resulting in increased infectivity of the virus and virus-related mortality [31].

Mukherjee et al. [3] determined more of these proviral interactions between RBPs and host miRNAs in sequence variations of SARS-CoV-2’s UTRs, identifying different possibilities of how mRNA-stabilizing RBPs can prevent miRNA-mediated RNA decay. Some binding sites for RBPs overlap with binding sites for host miRNA, preventing RISC binding and RISC-induced RNA decay. This is the case with miR-34b-5p and RBMS3 [3]. It is also possible that miRISC and an RBP compete for the same binding site, where the RBP might outperform the miRNA, as seen with miR-3664-5p and SRSF5. Furthermore, miRNA binding might be prevented by a specific nucleotide variation in an overlapping binding site, while RBP binding remains possible; an example of this scenario is seen in miR-9-5p and HNRNPA1 [3]. Mutations in the UTR sequence can therefore have an effect on viral fitness by changing or creating new miRNA or RBP binding sites, e.g., the creation of a new host miRNA binding site in a UTR is expected to enhance RNAi and weaken viral replication [3].

## 4. Factors Enhancing Viral Pathogenicity

### 4.1. Mutations in the Viral Genome

Mutations in the viral genome have a critical effect on the pathogenicity and susceptibility of the virus to the antiviral immune response, e.g., by changing the RNA secondary structure or creating new binding sites for host miRNAs [24]. Since binding sites for host miRNAs are expected to decrease viral fitness by making the virus more vulnerable to RNA interference, it is obvious that mutations leading to the creation of such will only withstand selective pressure if they do not cause a real disadvantage to viral replication and dissemination [16]. To give an example, a certain SARS-CoV-2 variant with a binding site for the human miR-4701-3p is only prone to miRNA-mediated RNA decay in limited amounts due to insufficient miR-4701-3p expression in lung tissue [3]. It is true for all mutations that in order to persist, they have to offer an advantage to the virus or at least not cause a disadvantage (with the exception of mutations located in highly conserved regions) [16]. Another example of this is the loss of miRNA binding sites through mutations, as seen in SARS-CoV-2 in the target of miR-197-5p, which was located in the NSP3 sequence. Due to the loss of this binding site, the miRNA is not able to bind and degrade viral transcripts anymore, sparing the virus from the miRNA-mediated immune defense. miR-197-5p is usually overexpressed in cardiovascular patients, who have a higher risk of mortality due to SARS-CoV-2 infection [24].

The mutation rate in RNA viruses is generally high because of the RNA-dependent RNA polymerase’s inadequate proofreading activity [16]. Nevertheless, the mutation rate of SARS-CoV-2 is reduced by the 30–50 exonuclease nsp14 in the RDRP complex, which also helps the virus to defend itself against the host’s base editor [24].

### 4.2. Influence of the Host miRNA Expression on Viral Pathogenicity

The susceptibility of a cell to virus infection is not only determined by its surface proteins but also by its miRNA expression pattern [16]. The most important proteins for cell invasion by SARS-CoV-2, comprising ACE2, TMPRSS2, and possibly disintegrin and metalloproteinase domain 17 (ADAM17) and furin, are all regulated by miRNAs. While TMPRSS2, ADAM17, and furin are co-receptors needed to complete the infection process [1], ACE2 serves as the receptor for SARS-CoV-2’s spike protein, enabling the virus to enter the host cell upon interaction with the S protein [6].

#### 4.2.1. Regulation of Receptor Expression

Lysine-specific demethylase 5B (JARID1B), which is encoded by the *KDM5B* gene, is responsible for the downregulation of several miRNAs targeting ACE2 and TMPRSS2, to the extent where, in the majority of human cells, ACE2 and TMPRSS2 are not expressed without the presence of JARID1B. Human respiratory epithelium cells show especially high expression levels of all three proteins [32]. The miRNAs directed against ACE2 and TMPRSS2 that are suppressed by JARID1B include hsa-let-7e/hsa-miR-125a [33] and hsa-miR-141/hsa-miR-200 [34].

Other miRNAs targeting TMPRSS2 include let-7a-g/i and miR-98-5p [35]. Let-7a-g/i, besides suppressing TMPRSS2 expression, also has an effect on immunity by regulating cytokine expression [1]. Let-7a-g/i is located in the intragenic region of a gene regulated by estradiol and is therefore upregulated after estrogen activation [35]. In contrast, all miRNAs of the let-7 family have been shown to be downregulated by androgens [36], providing one possible explanation for the gender disparities in the severity of COVID-19, which usually affects men more seriously than women [37]. miR-98-5p is another estrogen-responsive miRNA [38] that represses not only TMPRSS2, but also IL-6 expression [1].

The let-7 miRNA family can be bound by the lncRNA H19, resulting in the decreased availability of let-7 in the cell, making it more vulnerable to SARS-CoV-2 infection [1]. H19 is overexpressed in cancer cells [39], leading to the conclusion that these cells may be highly susceptible to virus infection [1]. Along with let-7, miR-145 and miR-222, which are directed against ADAM17 [1], are also suppressed in lung cancer cells [40], possibly leading to higher expression rates of TMPRSS2 and ADAM17, which again would make the cells more susceptible to virus infection [1]. While miR-222 is estrogen-dependent, just like the miRNAs targeting TMPRSS2 [35], miR-145 is upregulated by vitamin D [41], which might explain the correlation between vitamin D deficiency and the severe progression of COVID-19 [42].

Furin, which is expressed ubiquitously in pulmonary, hepatic, and intestinal tissue, is responsible for the cleavage of SARS-CoV-2’s S protein [43], which is important for membrane fusion during cell invasion [6]. Furin expression might also be regulated post-transcriptionally by miRNAs, namely, miR-20b, miR-19a, miR-19b, and miR-106a, which are all estrogen-dependent [1].

#### 4.2.2. Other Ways miRNAs Influence Susceptibility to Virus Infection

miRNAs play an important role in the secretion of the airway surface liquid (ASL) by regulating the ion channels and transporters responsible for the para- and transcellular movement of water and electrolytes [44]. The ASL covers the surface of epithelial cells in the respiratory tract, where one of the main functions is the protection of the host from inhaled pathogens, such as SARS-related coronaviruses [44,45,46]. Apart from airway surface liquid homeostasis, miRNAs have various other impacts on the immune defense of the respiratory tract [44].

Low expression levels of miRNAs targeting SARS-CoV-2, as seen in elderly patients, correlate with a higher risk of severe disease progression and mortality for COVID-19 [25].

### 4.3. Virus-Induced Alterations in the Transcriptome of the Host Cell

During virus infection, the transcriptome of a host cell, including miRNA and lncRNA expression patterns, is changed due to the initiation of the innate immune response in the infected cell. In addition to these host cell-induced developments, the virus may alter expression levels of host miRNAs by binding and destroying these molecules, potentially leading to an augmentation of cellular conditions for virus replication and dissemination [16]. Furthermore, the virus is able to synthesize miRNAs that interfere with cellular pathways by itself, enhancing its own pathogenicity and downregulating the host cell’s immune response [11]. Virus invasion also results in the alteration of siRNA expression profiles in the host cell and the generation of so-called “virus-activated siRNAs” (va-siRNAs), some of which might act as antivirals, while others might have proviral effects [47].

#### 4.3.1. Virus-Induced Changes of miRNA Expression

A study conducted by Mallick et al. [48] in 2009 evaluated the miRNA landscape in human bronchoalveolar stem cells (BASCs) during SARS-CoV infection, showing the upregulation of miR-17*, miR-574-5p, and miR-214, which repress virus replication and contribute to immune evasion until a successful transmission of the virus has taken place, as well as the downregulation of miR-223 and miR-98, which serves the regulation of BASC differentiation, activation of proinflammatory cytokines, and ACE2 suppression [48].

miRNAs can be used as biomarkers for the diagnosis of certain infectious diseases, e.g., miR-519c-3p serves as a biomarker to distinguish community-acquired pneumonia from chronic obstructive pulmonary disease exacerbations [49]. Guterres et al. [50] suggest that miRNAs may also be used as biomarkers for the determination of the disease progression of COVID-19. Suitable miRNAs could be directed at molecules that are responsible for the downregulation of inflammatory cytokines and chemokines since an increase in the expression levels of those miRNAs would result in enhanced production of proinflammatory cytokines during SARS-CoV-2 infection [50].

#### 4.3.2. The Role of lncRNA in the Cellular Response to Virus Infection

A transcriptome analysis of murine SARS-CoV-infected cells in 2010 by Peng et al. [51] uncovered around 500 annotated lncRNAs. Since the expressed lncRNAs were associated with type I interferon receptor and signal transducer and activator of transcription 1 (STAT1) and most were similarly regulated in the examined cells after infection with influenza virus and interferon treatment, it seemed likely to the authors that lncRNAs are involved in the regulation of the innate antiviral immune response of host cells [51]. In addition to these findings, another study by Josset et al. from 2014 [52] provided evidence for the co-expression of most virus infection-associated lncRNAs with genes involved in the lung homeostasis and immune response.

#### 4.3.3. lncRNA Expression in SARS-CoV-2-Infected Cells

In an effort to identify cellular pathways during SARS-CoV-2 infection, Vishnubalaji et al. [53] evaluated transcriptome data from normal human bronchial epithelial (NHBE) cells infected with SARS-CoV-2. An upregulation was observed in IFN-responsive gene targets leading to activation of the innate immune response. Interestingly, the NHBE cells showed overexpression of the lncRNA metastasis-associated lung adenocarcinoma transcript 1 (MALAT1), which is also known to be overexpressed in multiple neoplastic diseases, as well as inflammatory processes after lung transplants [53]. Since it has been shown that the silencing of MALAT1 mitigates inflammatory injuries after lung transplants by the inhibition of neutrophil chemotaxis [54], it might also lead to a reduction in the prevalence of cytokine storms in SARS-CoV-2 patients. Furthermore, the authors suggest that host-derived lncRNAs in SARS-CoV-2 infected cells, such as MALAT1 and nuclear-enriched autosomal transcript 1 (NEAT1), could potentially be used as biomarkers for infection [53].

#### 4.3.4. Expression of Viral miRNAs Mirroring Human miRNAs

As mentioned above, viruses may express miRNAs themselves, affecting pathogenicity via the downregulation of the host cell’s immune response or the creation of a proviral intracellular environment. Arisan et al. [11] identified seven sequences in the genome of SARS-CoV-2 that are completely identical to human miRNAs and evaluated the impact of the molecular pathways linked to these miRNAs on the pathogenesis of COVID-19 [11]. Their findings were that more than half of the seven miRNAs (including miR-8066, miR-3934-3p, miR-1307-3p, miR-1468-5p, and miR-3691-3p) were associated with the TGF-β signaling pathway [11]. TGF-β is a cytokine responsible for lung development and alveolarization, as well as the homeostasis and extracellular matrix composition of lung tissue. Additionally, it affects the immunity, survival, migration, and apoptosis of host cells [55]. Almost as many of the miRNAs (miR-8066, miR-5197, and miR-3934-3p) were involved in mucin-type O-glycan synthesis. The O- and N-glycosylation patterns of SARS-CoV-2’s S protein, where the latter of which is influenced by miR-8066 [11], are important for viral entry into the cell [56]. Further relevant effects of miR-8066 are related to the induction of the cytokine storm that may be observed in severely ill patients with COVID-19; the miRNA not only targets genes responsible for cytokine regulation [11] but its sequence also includes a core motif correlated with an increased probability of TLR-8 (toll-like receptor 8) expression via NF-κB, which leads to cytokine synthesis [57]. miR-3934-3p is also associated with the biosynthesis of heparan sulfate [11], which, as part of proteoglycans, serves as a binding site for SARS-CoV-2 on the host cell during the early attachment phase of virus invasion [58]. Additionally, miR-3934-3p is linked to vitamin assimilation [11], which might be interesting since vitamin D deficiency is associated with the increased severity of COVID-19 progression [42]. Other pathways related to the identified miRNAs included the cytochrome P450-mediated metabolism of xenobiotics, morphine addiction, semaphorin signaling, pulmonary hypertension, and cardiac fibrosis [11].

## 5. Non-Coding RNAs in Therapeutic Approaches

### 5.1. RNA Interference Using Artificial siRNAs

Although siRNA-mediated RNAi is a mechanism that is only present in plants and invertebrates, it may be induced therapeutically in humans, as well. For this purpose, a pre-synthesized siRNA is administered to the cell, where it binds specifically to complementary sequences as part of the RISC, which leads to post-transcriptional gene silencing [59]. Because siRNAs are only 21 to 23 bp in length, they are not able to activate the innate IFN immune response in the host cell, which may only be induced by dsRNAs larger than 30 bp [60]. Nevertheless, it is necessary to use siRNAs only in small doses, as high concentrations of siRNA have been observed to lead to undesirable effects, such as the induction of genes related to stress and programmed cell death [61]. Additionally, even though siRNAs are highly specific in general, there may be a certain amount of unintended gene silencing, where siRNAs may deter partially complementary mRNAs from being translated. This is problematic, as there will be partially complementary sequences in the human genome for most siRNAs [62].

Yet, in a study by Tang et al. [63] investigating anti-SARS-CoV siRNAs in rhesus macaques in 2008 via intranasal administration using a carrier, no siRNA-induced toxicity was observed. Instead, the authors proved the siRNA’s antiviral activity after administration resulted in fever relief and milder diffuse alveoli damage (DAD) [63].

In the last few years, siRNA therapeutics for various diseases have advanced to phase 3 of clinical trials [64]. One of them, namely, ONPATTRO^TM^ (patisiran), was approved for the treatment of patients with hereditary transthyretin-mediated amyloidosis in the USA and Europe in 2018 [65]. Another siRNA therapy that is under investigation is aimed at the treatment of the hepatitis B virus (HBV) and showed a strong reduction of the targeted HBV S antigen in human trials [66]. There have also been studies on siRNA therapeutics against SARS-CoV in the years following the initial SARS outbreak in 2002 ([67,68,69,70,71,72,73,74,75,76,77,78,79,80,81]; see below) and the progression of this research, which could potentially lead to the development of RNAi drugs against the novel SARS-CoV-2, is strongly endorsed.

### 5.2. Administration of siRNAs

There are two ways in which the desired effect of RNAi in the targeted cell may be provoked: on the one hand, the pre-synthesized siRNA may be transfected directly into the cell using a suitable carrier, while on the other hand, plasmid vectors encoding for shRNA, which will be processed to siRNA intracellularly, may be used. Both methods offer different advantages: while the administration of pre-made siRNA via viral vectors is highly efficient compared to the transfection of plasmid DNA, the latter option leads to gene-silencing for months after successful vector delivery. The transfection of siRNA, on the other hand, only induces a silencing effect for a few days because siRNA is degraded steadily after administration [59]. One approach to improve the serum half-life of artificial siRNA, while simultaneously reducing unspecific off-target effects, is the modification of siRNA with a locked nucleic acid (LNA), which is a synthetic nucleotide analog [82].

For the safe and efficient delivery of siRNA, carriers, such as lipid nanoparticles (LNPs), can be used, which protect the siRNA from enzymal degradation during administration and deliver it selectively to the targeted tissue [83,84]. LNPs may be administered intranasally for the treatment of diseases affecting the lung, such as COVID-19, and have been suggested as carriers for siRNA targeting SARS-CoV-2 by Itani et al. in a recent review [85]. Interestingly, cationic liposomes have been shown to have a greater bioavailability following intranasal administration than anionic ones, which is due to their electrostatic interaction with the negatively charged mucosa of the respiratory tract [86]. There are also several other ways to deliver RNAi therapeutics to the targeted tissue, including the use of exosomes ([87]; see below), natural or synthetic polymers, dendrimers, gold, magnetic iron oxide and silicia nanoparticles, quantum dots (QDs), carbon nanotubes (CNTs), or an N-acetylgalactosamine conjugated siRNA system (GalNAc-siRNA) [88,89,90].

### 5.3. Targeted Sequences in SARS-CoV

Ever since the outbreak of SARS-CoV in 2003, there has been a lot of research concerning siRNA-mediated RNA interference in SARS-CoV-infected cells. In most of these studies, the targeted sequences were those of the four structural proteins of SARS-CoV. These four major proteins comprise the N, M, S, and E proteins [67].

The nucleocapsid, or N protein, apart from forming the long helical nucleocapsid of the virus, also plays a role in RNA synthesis [67]. Effects of the N protein include the induction of the apoptotic pathway, upregulation of proinflammatory cytokine production, and inhibition of the antiviral response of the innate immune system. Additionally, it enhances the production of IFNβ [68], which is primarily increased by the M protein [69]. It has been reported that the targeting of the N gene in SARS-CoV has led not only to the reduced expression of the N gene but also to decreased IFNβ production [68].

The M (membrane) glycoprotein, apart from increasing IFNβ synthesis [68], is also essential for virus budding and assembly [70] and is highly abundant in infected cells [45]. In a study by Wang et al. from 2010 [69], two highly conserved regions in the RNA sequence encoding for the M protein were targeted by artificial siRNAs, which led to decreased expression of the M gene. The authors found out that the 5’ half of the M gene of SARS-CoV was seemingly more susceptible to spontaneous mutations than the 3’ half, which made them choose to target sequences in the latter [69].

The next important structural protein encoded by SARS-CoV is the S glycoprotein located on the viral capsule, which is responsible for the invasion of host cells [67]. The spike protein consists of two subunits: S1 binds to ACE2 on the host cell membrane and S2 mediates fusion between the cell membranes of the virus and the cell [71]. The S protein serves as an antigen for the specific antibody and T cell response of the host cell [72]. siRNAs directed against SARS-CoV’s S protein have been shown to successfully suppress the expression of the S protein, as well as the replication of the virus in infected cells [73,74]. siRNA duplexes targeting ORF1b in addition to the S protein have also been investigated in monkeys and have been shown to inhibit virus replication, mitigate SARS symptoms, and protect the lungs from harm [75]. Another study by Wu et al. [76] also proved the antiviral effect of siRNAs directed against the S protein and the 3’ untranslated region of SARS-CoV.

Lastly, the envelope, or E protein, is responsible for virus assembly and has been successfully targeted by siRNAs at two different sites by Meng et al. [77]. The same study also investigated different siRNAs directed against the gene encoding the RNA-dependent RNA polymerase (RDRP), where only two out of four siRNAs lead to a decrease in RDRP expression [77].

There have also been studies investigating siRNAs directed at two different structural genes, demonstrating that these siRNAs had even better antiviral effects than siRNAs only targeting one gene [78] and showing that the concentration of the siRNA duplexes correlated with antiviral activity [79]. Another study by Li et al. [80] evaluated siRNA targeting the leader sequence of SARS-CoV, reporting a much stronger inhibitory effect on virus replication than siRNA targeting the S protein gene.

Furthermore, it is possible to target subgenomic RNA translated from one of SARS-CoV’s 14 open reading frames with siRNA, as shown by Akerstrom et al. [81], who tested siRNA directed at sgRNAs 2, 3, and 7, resulting in lower viral reproduction. Moreover, Chen et al. [91] demonstrated that the transfection of siRNA-targeting ORF8a to a SARS-CoV infected cell led to a decrease of greater than 50% in the replication of the virus.

In contrast, siRNA directed at ORF3a did not lead to a decrease in replication after administration to an infected cell; however, it significantly suppressed the virus release. This was found out by Lu et al. [92] in an effort to identify the function of the ORF3a ion channel using siRNAs for gene silencing.

### 5.4. Attributes of Potential RNAi Targets in SARS-CoV-2

As of September 2020, no siRNAs targeted at sequences of SARS-CoV-2 have been tested yet. The characteristics of a potential target, however, remain clear: sequences targeted by siRNAs cannot be longer than 21 to 25 nucleotides, and they should not be similar to sequences in the human genome, since this could lead to unintended silencing of the host genes [93]. Furthermore, it is advisable to target only highly conserved regions of the viral RNA that have a low susceptibility to spontaneous mutations because if the virus acquires a mutation in the target site, the transcripts of the sequence in question may not be degraded by the RISC anymore, as there would be a lack of complementarity to the associated siRNA. The risk of RISC dysfunction due to virus mutation may also be lowered by using two or more different siRNAs simultaneously such that even if one complementary sequence mutates, the effect of the RISC will still be observed on the other [59].

Target sites may encode for proteins essential for viral replication, e.g., the RNA-dependent RNA polymerase, but also certain proteins encoded by the host cell DNA, which are adopted by the virus for its own reproduction [67]. Moreover, siRNA may also be directed against host genes that are necessary for viral entry to the cell [59]. For example, a study by Lu et al. from 2008 [94] used siRNA-targeting ACE2 mRNA, which led to the silencing of ACE2 expression and consequently reduced SARS-CoV infection in the transfected Vero E6 cells.

### 5.5. Viral Suppression Strategies of RNAi

Viral RNAi-suppressor proteins prevent the degradation of viral RNA by inhibiting the generation of siRNA and the RISC assembly of existing siRNA [13]. According to Karjee et al. [95], one of the viral proteins acting as RNA silencing suppressors in SARS-CoV is derived from ORF7a. It is a transmembrane protein that is localized mainly in the ER and Golgi of the host cell, where the viral genome is replicated [47]. The reason for the localization of the 7a protein is that in replication, dsRNA is generated, which is the main trigger of (natural) siRNA production [96]. Another important RNAi suppressor protein is SARS-CoV’s structural nucleocapsid (N) protein [13]. Because of the homology of the two viruses, it is likely that the 7a and N protein also act as RNAi suppressors in SARS-CoV-2, which would certainly be an interesting and potentially promising direction for future research. The downregulation of these proteins might be achieved using artificial siRNAs or CRISPR-Cas13a [47].

### 5.6. miRNA-Related Approaches

miRNAs could potentially be used therapeutically in gene therapy vectors, vaccines, or as antiviral drugs [16]. To give an example, Ivashchenko et al. [97] suggest the use of artificial complete complementary miRNA (cc-miR) that is able to bind to the gRNA of SARS-CoV-2. The cc-miR, which is coated by vesicles, could be administered specifically to the lung via inhalation, or introduced to the blood, which would lead to antiviral effects in every tissue the virus is able to enter. The authors designed a cc-miR based on miR-5197-3p, which interacts effectively with the gRNA of SARS-CoV-2 but also has binding sites in human genes. To prevent off-target effects, the artificial cc-miR was designed to have only low complementarity to these human target sites [97].

Regarding the significance of miRNAs for vaccines, Hosseini et al. [24] also suggest that the inclusion of binding sites for host miRNA into the viral genome could be a means to debilitate the live viruses used for active vaccination.

Another way in which synthetic miRNAs could be used as a potential therapy or vaccine against SARS-CoV-2 is proposed by Kreis et al. [98] and is related to the adaption of a natural antiviral mechanism in the human placenta: trophoblasts secrete exosomes comprising miRNAs of the C19MC (chromosome 19 miRNA cluster) in order to transfer their antiviral effects to other placental cells, as well as maternal and fetal cells. These miRNAs include miR517-3p, miR516b-5p, and miR512-3p, which all have an inhibitory effect on both RNA and DNA viruses and induce autophagy of cytoplasmic viruses in infected cells [98].

Serum-derived exosomes have several advantages over other sRNA delivery systems: they hold the potential to specifically target certain cell types and, since they are secreted endogenously, they are less likely to elicit undesired immune responses in the host [87].

This was also proven in a study by Zhang et al. [99], who successfully delivered miRNA and siRNA-packed exosomes to alveolar macrophages via intratracheal administration in a murine model. The predicted effects of the sRNAs were observed in the macrophages, but no anti-exosome immune response was provoked. To achieve the delivery of exosomes to epithelial and other lung cells, a method preventing their uptake by macrophages is needed [99].

Furthermore, Chow et al. [100] state that the expression rates of certain miRNAs targeting SARS-CoV-2 are very low in lung epithelia, which makes these tissues especially vulnerable to infection, and suggest that by therapeutically increasing the abundance of those miRNAs in respiratory epithelial cells, the antiviral defense mechanisms of the cells may be enhanced.

## 6. Conclusions

Non-coding RNAs are involved in various and complex mechanisms in SARS-CoV and SARS-CoV-2 infection, many of which have yet to be fully understood.

During infection, the viruses change the host’s miRNAome to augment cellular conditions for their own replication and assembly, while encoding for miRNAs that interfere with cellular pathways themselves. On the other hand, non-coding RNAs, including miRNAs, play an essential role in antiviral immunity by regulating the expression of cellular receptors for virus invasion and forming RISCs with proteins that may degrade, and therefore, silence viral RNA. Mutations in the viral genome affecting miRNA binding sites may enhance pathogenicity by enabling the virus to evade RNA interference. Inducing siRNA-mediated RNAi by transferring artificial siRNAs complementary to viral RNA sequences to infected cells is one promising approach for curing SARS-CoV-2 infections using non-coding RNAs. Further development of this idea requires more research on possible targets of siRNA in the viral genome. Other antiviral treatments that utilize miRNAs, e.g., as gene therapy vectors or in vaccines, have also been suggested.
